# Multi-Objective Optimizations for Microinjection Molding Process Parameters of Biodegradable Polymer Stent

**DOI:** 10.3390/ma11112322

**Published:** 2018-11-19

**Authors:** Hongxia Li, Kui Liu, Danyang Zhao, Minjie Wang, Qian Li, Jianhua Hou

**Affiliations:** 1 School of Mechanical Engineering, Dalian University of Technology, Dalian 116023, China; hxli@dlut.edu.cn (H.L.); Lkui@mail.dlut.edu.cn (K.L.); danyangz@163.com (D.Z.); mjwang@dlut.edu.cn (M.W.); 2 National Center for International Joint Research of Micro-Nano Molding Technology, Zhengzhou University, Zhengzhou 450000, China; houjianhua@zzu.edu.cn

**Keywords:** polymeric stent, injection molding, residual stress, warpage, kriging surrogate model, design optimization

## Abstract

Microinjection molding technology for degradable polymer stents has good development potential. However, there is a very complicated relationship between molding quality and process parameters of microinjection, and it is hard to determine the best combination of process parameters to optimize the molding quality of polymer stent. In this study, an adaptive optimization method based on the kriging surrogate model is proposed to reduce the residual stress and warpage of stent during its injection molding. Integrating design of experiment (DOE) methods with the kriging surrogate model can approximate the functional relationship between design goals and design variables, replacing the expensive reanalysis of the stent residual stress and warpage during the optimization process. In this proposed optimization algorithm, expected improvement (EI) is used to balance local and global search. The finite element method (FEM) is used to simulate the micro-injection molding process of polymer stent. As an example, a typical polymer vascular stent ART18Z was studied, where four key process parameters are selected to be the design variables. Numerical results demonstrate that the proposed adaptive optimization method can effectively decrease the residual stress and warpage during the stent injection molding process.

## 1. Introduction

Polymeric stents are a promising prospect [1]. However, the thin-walled tubular surface of polymeric stents has a discontinuous mesh structure. Furthermore, the stent has a tiny entirety, big length-to-diameter ratio, partial precision and unique structure, which greatly limits the machining and application of polymeric stents. The traditional machining method for stents is laser cutting. However, there are some uncontrolled machining problems, such as cutting seam width, surface roughness, taper of cut, back injury, slag, recast layer, etc. Fortunately, Clarke et al. presented an innovative technique for manufacturing polymer stents through the injection molding process [2]. This method provides a new line of thought for the high efficiency machining of stents. The injection molding stents have many remarkable advantages such as its strong reproducibility, high molding efficiency, good surface quality, good material condensation orientation, good molding consistency, etc.

At present, the injection molding method is not widely used in stent manufacturing. An important cause is that the parameters greatly influence molding quality of the polymeric stent during the injection molding process. Moreover, it is hard to determine a reasonable process parameter combination for the injection molding process, and this can easily result in some problems. For example, the flow of the melt is difficult to manage or the melt flows unevenly, it is hard to fill the mold cavity, the relatively large residual stress in the stent’s cavity results in difficulty in demolding, and the stent undergoes a warpage phenomenon after demolding. Therefore, there is a need to investigate the effect of some important process parameters, such as mold temperature, melt temperature, flow rate and packing pressure, on stent quality. A reasonable process parameter combination is able to ensure the molding quality of the polymeric stent. Some scholars have conducted studies on the effect of injection molding process parameters on product quality. Liu et al. studied how the mold temperature affected the shrinkage of rapid heat cycle injection molded parts [3]. Duo performed the Taguchi experiment to investigate the effect of the injection molding process parameters, such as packing time, molding temperature and cooling time, on the warpage of flat tiny devices [4]. Jiang et al. revealed the great influence of mold temperature on the structure and mechanical properties of microinjection molding PP products [5]. In the meantime, Singh et al., Annicchiarico et al. and Mohan et al. conducted a detailed study of the effect of injection molding process parameters on the quality of injection molding products, providing a reference value for the stent injection molding process [6,7,8]. The above-mentioned studies were based on the effect between process parameters and product quality indicators, and some of these scholars were able to establish a function relationship between process parameters and quality indicators from the perspective of the optimal design. Based on the combination of the artificial neural network and genetic algorithm, Shen et al. optimized the injection molding process parameters, and improved the volume shrinkage of parts [9]. Based on the software tools, Florin et al. optimized the microinjection molding process of polymer medical apparatuses and instruments [10]. Kitayama et al. realized the multi-objective optimization of volume shrinkage and clamping force in the plastic injection molding process through the sequential approximate optimization method [11]. Based on Taguchi, ANOVA and the artificial neural network method, Oliaei et al. investigated the warpage and shrinkage optimization of biodegradable polymer injection molding plastic spoon parts [12]. Dang proposed a general framework for optimizing injection molding process parameters [13]. Kashyap et al. presented a detailed summary of related methods for the optimization of injection molding process parameters [14]. Kitayama et al. performed a multi-objective optimization of process parameters in plastic injection molding for simultaneously minimizing the warpage, cycle time and clamping force using radial basis function [15]. These studies have laid a solid foundation for the process parameter control of the stent injection molding process.

However, the polymeric vascular stent has a thin-walled tubular network structure with micro-scale geometry (strut width and thickness of 0.1 mm), large ratio of length to diameter and local precision, which make molding quality very sensitive to changes in process parameters. Furthermore, the relationship between process parameters and molding quality is very complicated, non-linear, implicit and multimodal. In addition, the number of functional assessments is extremely time consuming. In this case, if using a conventional optimization method (such as a gradient-based algorithm), it will be difficult to get a global optimization design for the process parameters. Here, it is worth recommending that the surrogate modeling, which mainly consisting of the Kriging method, can effectively solve the engineering problems mentioned above [16]. It is well known that the microscopic properties of polymer materials affect macroscopic properties, but the relationship is complex, and the computational process is highly time-consuming [17]. Fortunately, one of the great benefits is that the surrogate model actually calculates material properties at the micro level, which can save the computation time. The surrogate model can establish a suitable approximation functional relationship between process parameters (input) and molding quality (output), thus taking the place of the complicated engineering calculation process at lower computational cost. In this respect, Gao et al. [18], Li et al. [19] have done a lot of related work. Gao et al. used Surrogate-based process optimization for reducing warpage in macro-injection molding. Li et al. have developed a multi-objective optimization based on the Kriging method. The method was applied to optimize the geometry of the biodegradable polymer stent, which finally showed that the stent expansion performance could be successfully improved.

In the present paper, the multi-objective process optimization about process parameters and molding quality was researched by constructing the adaptive optimization algorithm based on Kriging surrogate modeling. The Kriging model was applied to establish an approximate functional relationship between stent molding quality (residual stress and warpage) and injection molding parameters, thus making it possible to avoid the time-consuming finite element reanalysis in microinjection molding process optimization. The optimization iterative process is based on approximate functional relationships to decrease the computation time. Meanwhile, the 40 trial sample points were obtained by using the Optimal Latin Hypercube Sampling Method (Optimal LHS) [20]. Also, the expected improvement (EI) function plays a huge role when performing the adaptive optimization process. Even in the case of small sample sizes, the local and global searches can also be balanced, making it more likely to find global optimization designs. The finite element method based on MOLDFLOW (Autodesk Moldflow Insight 2012, Autodesk Inc., San Rafael, CA, USA) was ran simultaneously to model the injection molding process of the stent to obtain the response of molding quality under the specific process parameter combination. The combination of the numerical results and the design optimization method of the study facilitates further optimization studies and development of a method for further manufacturing a polymer stent by a microinjection molding process.

## 2. Materials and Methods

### 2.1. PLA Stent Material

Biodegradable polymeric stents are mainly made of polylactic acid (PLA) and modified materials. In the present study, as an example, the biodegradable polymeric stent was made of semi-crystalline PLA (Manufacturer: Kao Corporation, Trademark: ECOLA S-1010, Tokyo, Japan), with a solid particle density of 1.6416 g/cm^3^ and a melt density of 1.4836 g/cm^3^.

**Figure 1:**
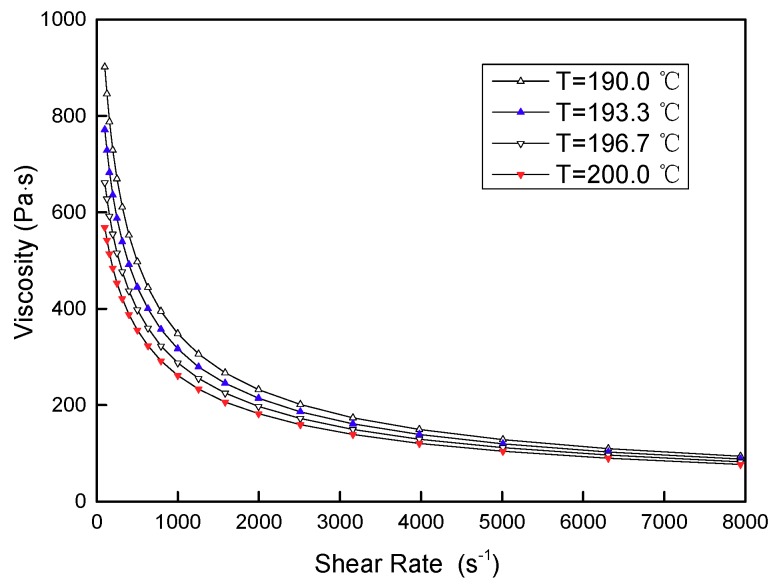
The rheological properties of polylactic acid (PLA).

The melt rheology curve of PLA is presented in Figure 1. On one hand, the flow process of the PLA melt under constant temperature exhibits a significant shear thinning behavior. In the actual injection molding process, and within the allowable shear rate of the material, this rheology should be fully used in selecting an appropriate melt flow shear rate, adjusting the melt viscosity, and improving flowability, thereby reaching a better flow and filling ability. On the other hand, the shear viscosity of the PLA melt was correlated to temperature. Within a certain range of shear rate, as a whole, viscosity decreases as temperature increases. Furthermore, the effect of temperature on viscosity can basically be ignored when the melt flow exceeds a certain shear rate. As a result, overall consideration should be given to the effect of temperature and shear rate on melt flowability for full utilization.

**Figure 2:**
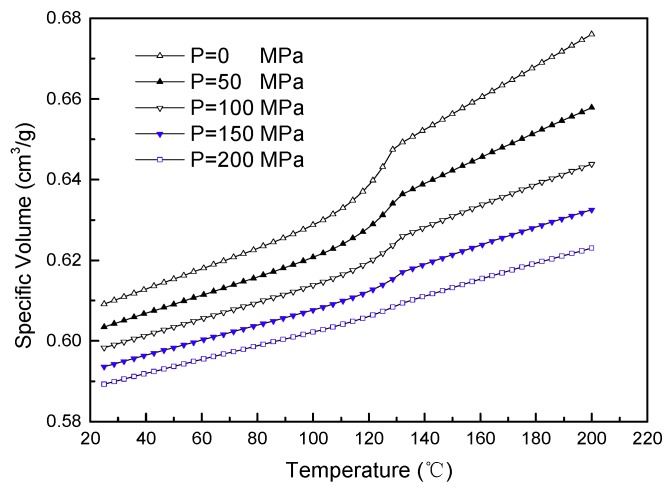
The PVT properties of polylactic acid (PLA).

The PVT curve of PLA plastic is presented in Figure 2 to investigate the relationship between pressure, volume and temperature. Under specific pressure conditions, the volume ratio of PLA increases as temperature increases. When pressure is relatively small, and as the temperature increases, PLA is converted from a solid particle status into a melt status, and the relationship between the volume and temperature of PLA tends to undergo a staged linear change. Furthermore, there will be a small upward slope near 130 °C. This is because PLA is a semi-crystalline material, and 130 °C is the crystalline transition temperature. The molecular chain of PLA is converted from a partially ordered arrangement status into an overall disordered status, which results in a relatively big volume expansion. The temperature drop led to significant volume shrinkage. In the injection molding process, consideration should be given to the effect of the material’s crystalline. When the pressure is very high, and the temperature increases, a few upward slopes under the crystalline transition temperature appear. This is due to the very high pressure at this moment, which greatly inhibits the movement of the molecular chain and the transition of material volume, resulting in the single linear change of the whole process. Therefore, it is necessary to flexibly control the injection pressure and the injection temperature due to the characteristics of PLA as a semi-crystalline material when performing the actual injection molding process.

### 2.2. Biodegradable Polymeric Stent Structure

**Figure 3:**
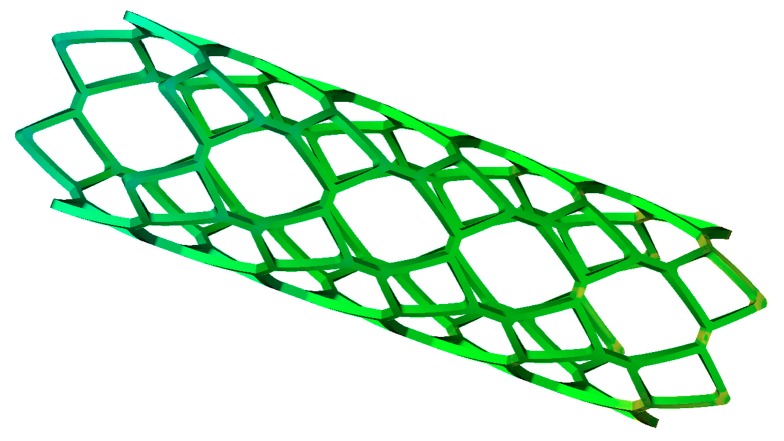
Generic polymeric stent with straight bridges.

The biodegradable polymeric stent made of PLA has good biocompatibility and a relatively short biodegradation period. The structure of this stent is presented in Figure 3: overall length, 13.75 mm; thickness, 0.17 mm; outside diameter, 3.36 mm.

It can be observed that it has a typical thin-walled structure and a large number of discontinuous mesh structures. Full consideration should be given to demolding properties and post-demolding warpage.

### 2.3. Microinjection Molding Optimization of Biodegradable Polymeric Stents

In the PLA stent injection molding process, due to its thin-wall and mesh structure feature, its common phenomena, such as demolding difficulty and post-demolding warpage, greatly influence the efficiency and molding quality of the stent injection molding process. Its demolding process is mainly influenced by the residual stress of the stent in the orientation direction, indicating the stress of parts before ejection, instead of after ejection. An excessively large residual stress in the cavity would cause the stent in the cavity to maintain a relatively high-tension status, which is not good for the demolding process. Warpage is a size deformation caused by the unbalanced change of inner stress. Serious warpage can even influence the normal use of stents. The residual stress and warpage of stents are influenced by processes, such as mold temperature, melt temperature, flow rate and packing pressure. Hence, a smaller residual stress and warpage would be better. Therefore, the four process parameters, that is, mold temperature, melt temperature, flow rate and packing pressure, should be viewed as design variables, while the two evaluation indicators, that is, residual stress of the stent in the orientation direction in the cavity (affecting the stent demolding process) and warpage (affecting the use properties of the stent), should be viewed as objective variables. Consequently, the multi-objective optimization design for the molding quality optimization problem during the stent injection molding process can be defined as follows:

(1) $$\begin{array}{ll} \min & \mathrm{residual}\;\mathrm{stress}(\mathrm{RS}),\;\mathrm{warpage}(W)\\ s.t. & x_{1}\leq x\leq x_{2} \end{array},$$

Residual stress refers to the residual stress of the stent in the cavity in the first principal direction. Warpage refers to the total warpage of the stent, where x represents the optimization variables, and $x_{1}$ and $x_{2}$ represent the upper and lower limits of these optimization variables.

Residual stress and warpage are the two main indicators for stent injection molding quality. However, the RS and W of a polymeric stent varies with significantly different scales, making it difficult to define a suitable weight. Naturally, if both of these are scaled to (0, 1), the weight can be assigned an intermediate value of 0.5. In this case, the optimization problem mentioned above can be written as:

(2) $$\begin{array}{ll} \min & f(x)=0.5\frac{\mathrm{RS}-{\mathrm{RS}}_{\min}}{{\mathrm{RS}}_{\max}-{\mathrm{RS}}_{\min}}+0.5\frac{W-W_{\min}}{W_{\max}-W_{\min}}\\ s.t. & x={\left[\begin{array}{cccc} T_{mold} & T_{melt} & v_{flow} & P_{pack} \end{array}\right]}^{T}\\ & 80\;{}^{\circ}C\leq T_{mold}\leq 90\;{}^{\circ}C\\ & 190\;{}^{\circ}C\leq T_{melt}\leq 205\;{}^{\circ}C\\ & 0.13\;{\mathrm{cm}}^{3}/s\leq v_{flow}\leq 0.25\;{\mathrm{cm}}^{3}/s\\ & 75\%\leq P_{pack}\leq 90\% \end{array},$$

where RS represents the residual stress of the stent in the cavity in the first principal direction and W is the total warpage. $T_{mold}$ is the temperature of the mold which can cool the melt, $T_{melt}$ is the melt temperature and has a large influence on the melt flow viscosity, $v_{flow}$ is the volume flow rate affecting the molding cycle, and $P_{pack}$ is the packing pressure.

### 2.4. Finite Element Method of the PLA Stent Injection Molding Process

**Figure 4:**
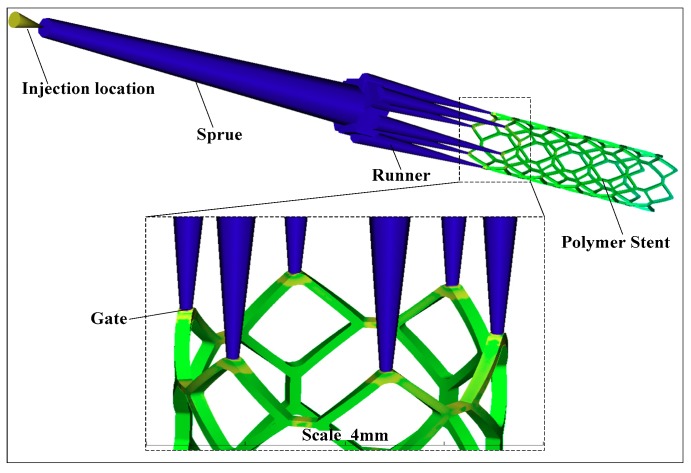
The injection molding process of stent based on the MOLDFLOW.

The stent microinjection molding process can be researched by using the finite element method (FEM). The numerical simulation platform based on the MOLDFLOW injection molding process was designed with a stent injection molding runner system, which includes six injecting gates (Figure 4). The runner system uses the cylinder units to divide the grids, which includes 501 cylinder units and 502 nodes, with an injecting system volume of 0.109295 cm^3^. The stent structure has a uniform wall thickness. Hence, it uses the double-layer grid division, which includes 80,670 triangular units and 40,227 nodes, with a volume of 0.00333709 cm^3^ and a grid matching ratio of 92.8%.

**Figure 5:**
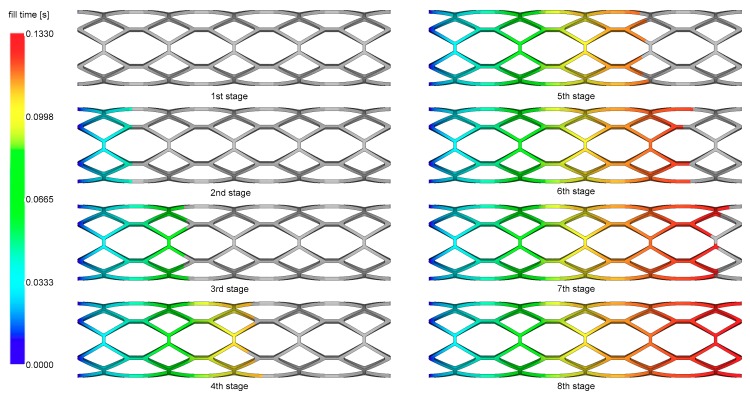
The injection molding process of a stent.

The injection molding runner system was adopted to conduct the numerical simulation of the stent injection molding process. The melt filling process is presented in Figure 5. The polymer melt is injected slowly into the cavity from the left end shown in Figure 5 and eventually fills the entire cavity. Compared to the eight filling stages, the filling process along the longitudinal direction is uniform.

### 2.5. Optimization Algorithm

#### 2.5.1. Approximate Method

The kriging model is an approximation technique that consists of a regression and a non-parametric part, including a polynomial and a random distribution:

(3) $$\hat{y}(x^{i})=F(\beta ,x^{i})+z(x^{i})=f^{T}(x^{i})\beta +z(x^{i}),$$

in which, β is the regression coefficient; $f(x^{i})$ is a polynomial that provides a global approximation of the simulation; and $z(x^{i})$ is a randomly distributed error that provides an approximation of the simulated local deviation and has the following statistical properties:

(4) $$\begin{array}{c} E\left[z(x)\right]=0\\ \mathrm{var}\left[z(x)\right]=\sigma_{z}^{2}\\ \mathrm{corr}\left[z(x^{i}),z(x^{j})\right]=R(\theta ,x^{i},x^{j})=\overset{m}{\underset{l=1}{∐}}\exp\left[-\theta {\left(x_{l}^{i}-x_{l}^{j}\right)}^{2}\right] \end{array}$$

where $x^{i}$ and $x^{j}$ are any two points in the training samples; $R(\theta ,x^{i},x^{j})$ is the correlation function with parameter θ, characterizing the spatial correlation between the points of training samples, and in this paper, Gaussian correlation function was used, which can be described as:

(5) $$R(\theta ,x^{i},x^{j})=\exp(-{\sum_{l-1}^{n}\theta_{k}\left|x_{l}^{i}-x_{l}^{j}\right|}^{2})$$

where *n* is the number of design variables; $x_{l}^{i}$ and $x_{l}^{j}$ are the *l*-th component of the training sample.

#### 2.5.2. Predictor

Given the training samples $S=[x_{1},x_{2},\ldots ,x_{n}]$ and corresponding response $Y=[y_{1},y_{2},\ldots y_{n}]$, the response at a new point $x^{*}$ can be expressed by a linear combination of Y

(6) $$\hat{y}(x^{*})=c^{T}Y,$$

The error is

(7) $$\hat{y}(x^{*})-y(x^{*})=c^{T}Y-y(x^{*}),$$

Substituting Equation (3) into Equation (7) gives

(8) $$\begin{array}{ll} \hat{y}\left(x^{*}\right)-y\left(x^{*}\right) & =c^{T}\left(F\beta +Z\right)-\left(f{\left(x^{*}\right)}^{T}\beta +z\right)\\ & =c^{T}Z-z+{\left(F^{T}c-f\left(x^{*}\right)\right)}^{T}\beta \end{array},$$

where $Z=\left[z_{1,}z_{2},\ldots ,z_{n}\right]$, $F=\left[f_{1},f_{2},\ldots ,f_{n}\right]$. To get the unbiased predictor for $x^{*}$, the mean error at this point should be zero, i.e.,

(9) $$E\left(\hat{y}\left(x^{*}\right)-y\left(x^{*}\right)\right)=0,$$

And then, $F^{T}c\left(x^{*}\right)-f\left(x^{*}\right)=0$. Therefore, we have the mean squared error of the predictor (8),

(10) $$\begin{array}{cc} \varphi \left(x^{*}\right) & =E\left[{\left(\hat{y}\left(x^{*}\right)-y\left(x^{*}\right)\right)}^{2}\right]\\ & =\sigma^{2}\left(1+c^{T}Rc-2c^{T}r\right) \end{array},$$

where

(11) $$r\left(x^{*}\right)=\left[R\left(\theta ,x_{1},x^{*}\right),\ldots R\left(\theta ,x_{n},x^{*}\right)\right],$$

It represents the spatial correlation between new sample $x^{*}$ and training samples. $c(x^{*})$ can be obtained by minimizing $\varphi (x^{*})$, i.e.,

(12) $$\begin{array}{l} c=R^{-1}\left(r-F\tilde{\lambda }\right)\\ \tilde{\lambda }={\left(F^{T}R^{-1}F\right)}^{-1}\left(F^{T}R^{-1}r-f\right) \end{array},$$

then

(13) $$\hat{y}\left(x^{*}\right)=f\left(x^{*}\right)\hat{\beta }+r{\left(x^{*}\right)}^{T}\gamma ,$$

where

(14) $$\gamma =R^{-1}\left(Y-F\hat{\beta }\right),$$

Thus, we can predict the function value $\hat{y}\left(x^{*}\right)$ at every new point $x^{*}$ by using Equation (13).

#### 2.5.3. Expected Improvement (EI)

Maximizing expectation improvement is an aspect of the method that adds new points that consider the prediction value and the prediction variance. Expected improvement (EI) calculates the probability of a target’s response improvement at a given point. Y(x) at some point x is unknown before sampling at this point. However, kriging can predict its mean $\hat{y}(x)$ and variance $\sigma^{2}$. Assuming that the response value of the current optimal design is $Y_{\min}$, the improvement of the response of this point is $I=Y_{\min}-Y(x)$ with a normal distribution. Thus, its probability density function is

(15) $$\frac{1}{\sqrt{2\pi }\sigma (x)}\exp\left[-\frac{{\left(Y_{\min}-I-\hat{y}(x)\right)}^{2}}{2\sigma^{2}(x)}\right],$$

Therefore, the expected improvement of the response value is

(16) $$E[I(x)]=\int_{I=0}^{I=\infty }I\left\{\frac{1}{\sqrt{2\pi }\sigma (x)}\exp\left[-\frac{{{(Y}_{\min}-I-\hat{y}(x))}^{2}}{2\sigma^{2}(x)}\right]\right\}dI,$$

Integrating Equation (16) by parts, it gives

(17) E[I(x)]=σ(x)[uΦ(u)+φ(u)],

where

(18) $$u=\frac{Y_{\min}-\hat{y}(x)}{\sigma (x)}$$

and where Φ and φ are the normal cumulative distribution and density functions, respectively. 

#### 2.5.4. The Convergence Criterion

The convergence condition of EI is

(19) $$\frac{EI(x)}{Y_{\max}-Y_{\min}}<\varepsilon_{1},$$

where $\varepsilon_{1}$ is the convergence tolerance. $Y_{\max}$ and $Y_{\min}$ are the maximal and minimal function values in samples, respectively.

In addition, the predicted value converges to the numerical result to characterize the accuracy of kriging model,

(20) $$\left|f(x_{l})-{\hat{y}}_{l}\right|<\varepsilon_{2}$$

where $f(x_{l})$ is the numerical result; ${\hat{y}}_{l}$ is the predicted value based on kriging; $\varepsilon_{2}$ is the convergence tolerance.

**Figure 6:**
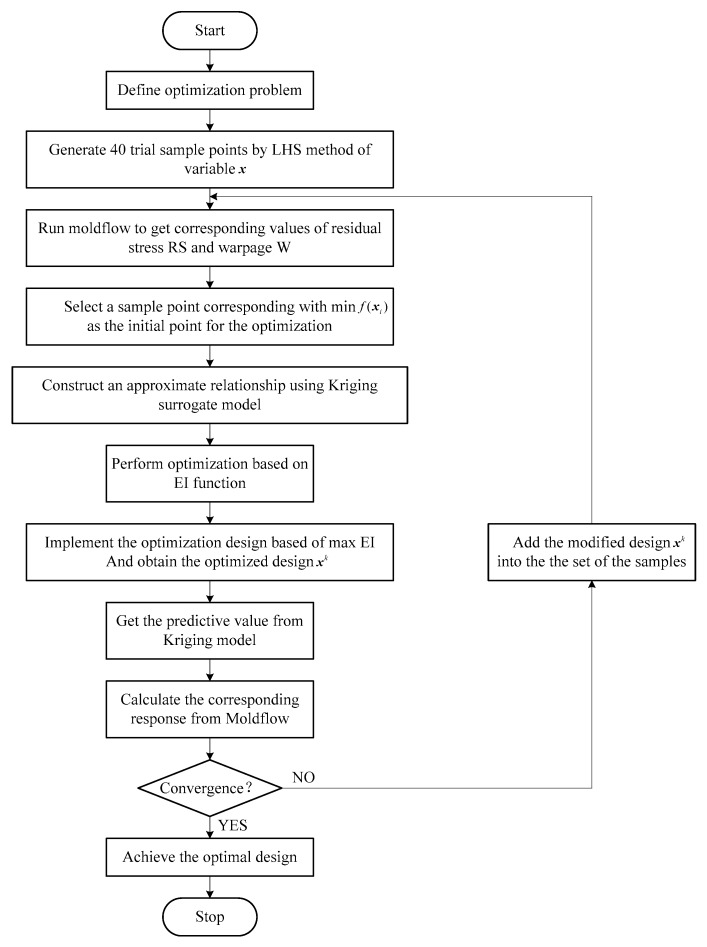
Complete process of the optimization algorithm based on the Kriging surrogate model.

Moreover, the optimization results of the last two iterations should be similar,

(21) $$\left|f(x_{l})-f(x_{l-1})\right|<\varepsilon_{3}$$

where $f(x_{l})$ and $f(x_{l-1})$ are the response values of the last two iterations, respectively. Figure 6 shows the complete process of the optimization algorithm based on the Kriging surrogate model, making this process easier to understand.

## 3. Results

### 3.1. Results of Stent Injection Molding Process

The boundary values of the four design variables were respectively given. Then, 40 initial training sample points were generated using the orthogonal LHS method to build the Kriging surrogate model, with the aim of establishing the approximate relationship between the two objective variables of the stent (residual stress and warpage) and the four design variables.

The design variables included mold temperature $T_{mold}$, melt temperature $T_{melt}$, flow rate $v_{flow}$, and packing pressure $P_{pack}$ and the range of the four process parameters were shown in Table 1.

**Figure 7:**
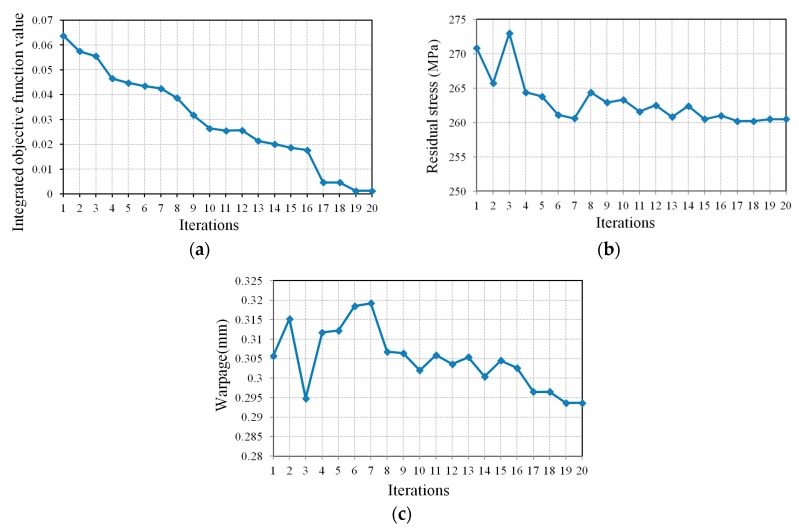
Optimization iterative process. (**a**) Relationship between integrated objective function value and iterations; (**b**) Relationship between residual stress and iterations; (**c**) Relationship between warpage and iterations.

The Kriging surrogate model optimization process needs 20 iterations to obtain the optimal solution corresponding to the minimum values of objective functions. The optimization iterative process is shown in Figure 7.

The comparison between the optimal design result and comparable design result is presented in Table 2. The comparable design results, which adopt the intermediate values of the boundary values of the four design variables recommended by MOLDFLOW, correspond to the residual stress and warpage results.

It can be observed that merely the design recommended by MOLDFLOW could not effectively reduce the residual stress and warpage of the stent, while the optimal design based on the iteration process can significantly reduce residual stress and warpage. Compared to the results of the comparable design, residual stress decreased by 20.3% and warpage decreased by 44.6%. Apparently, this was attributable to the enhancement of the molding quality of the stent.

**Figure 8:**
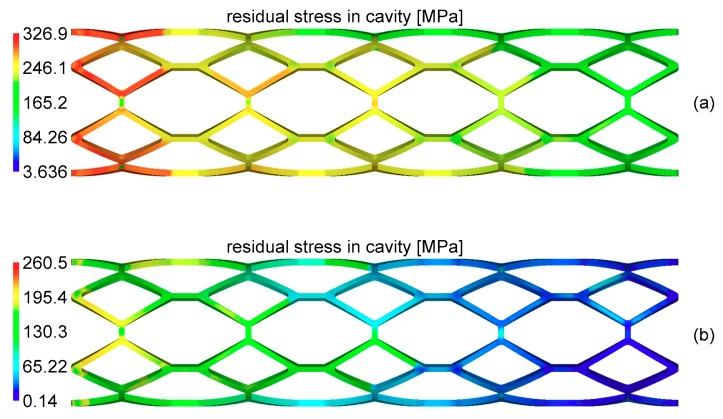
The distribution of residual stress of the comparable design and optimal design. (**a**) The distribution of residual stress of the comparable design; (**b**) The distribution of residual stress of the optimal design.

**Figure 9:**
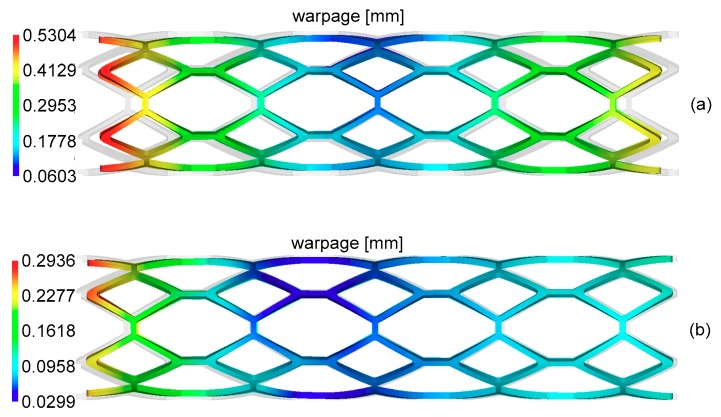
The distribution of warpage of the comparable design and optimal design. (**a**) The distribution of warpage of the comparable design; (**b**) The distribution of warpage of the optimal design.

Figure 8 and Figure 9 show the corresponding residual stress and warpage of these two kinds of designs, and the results of the optimal design obviously had smaller residual stress and warpage.

### 3.2. Results of Single Factor Analysis

In order to investigate the influences of these four important parameters on the residual stress and warpage of the stent during the injection molding process, it is necessary to carry out a single factor analysis. In the optimal process combination, the influence of the remaining factor on the result was analyzed by fixing three factors, and the results are shown as follows. It revealed that the effects and mechanisms of these four parameters on the residual stress and warpage of the stent are significantly different.

**Figure 10:**
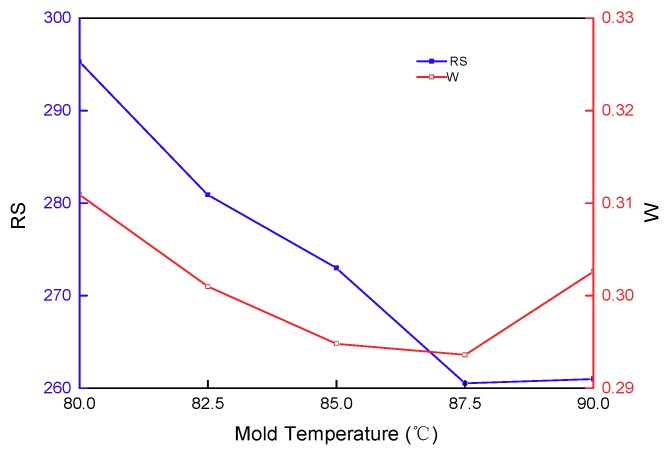
The effect of mold temperature on the residual stress and warpage.

The effect of mold temperature on the residual stress and warpage of the stent is presented in Figure 10. As illustrated by the curve change trend, mold temperature has a relatively significant influence on the residual stress and warpage of a stent. Raising the mold temperature could decrease the warpage. As for the PLA semi-crystalline material, high mold temperatures can reduce the flow viscosity of the melt, causing the melt to easily flow and enhance mold-filling efficiency. A relatively high mold temperature can decrease the temperature difference of the melt, cool the melt uniformly, reduce shrinkage deformation, and prevent defects, such as depression, deformation, etc. Excessively high mold temperatures and being close to the boundary value expands the volume of the melt, causes warpage, and prolongs the cooling time, thereby reducing production efficiency. Raising the mold temperature is attributable to improving surface roughness, reducing inner stress and the degree of orientation, enhancing the strength of the weld line and product density, which thereby reduces the impact strength of the streamline direction, and reduces residual stress in the cavity.

**Figure 11:**
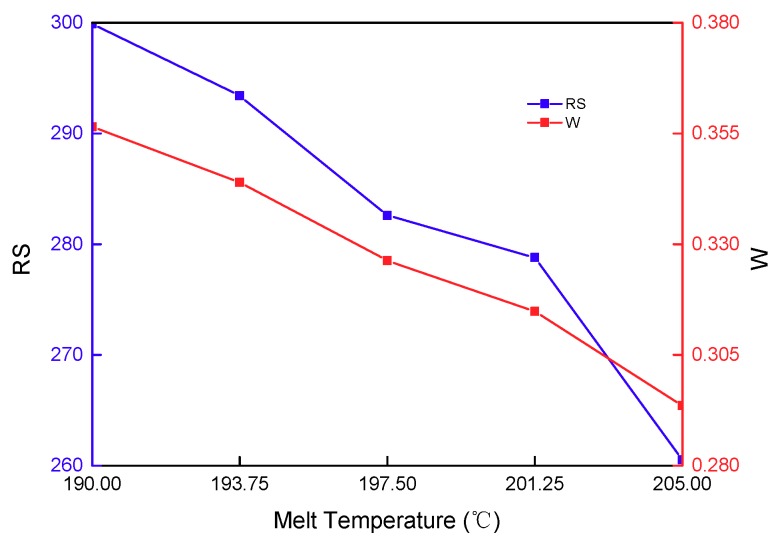
The effect of melt temperature on the residual stress and warpage.

The effect of melt temperature on the residual stress and warpage of a stent is presented in Figure 11. As for the PLA semi-crystalline material, with its small crystalline tendency, the melt temperature would mainly affect the viscosity, flowability and molecular orientation of the melt. On one hand, enhancing the melt temperature would expand the volume of the melt, reducing the amount of material into the cavity. Furthermore, this would cause the loose status of the polymer in the orientation direction, and enhance the molecular chain orientation ability, resulting in an increase in warpage. On the other hand, enhancing the melt temperature can reduce the viscosity of the melt, and enhance the shear rate of the melt shear rate, when the injection pressure and packing pressure remain unchanged. This is attributable to the transfer of the pressure to the cavity, reducing the warpage. In general, as for the latter, viscosity has a relatively significant influence on temperature-sensitive materials. As for the former, viscosity has a relatively significant influence on non-temperature-sensitive material. PLA melt viscosity is relatively sensitive to temperature. Therefore, enhancing the melt temperature, as a whole, can reduce warpage. Furthermore, enhancing the melt temperature can effectively reduce melt viscosity. This would cause filling to be more uniform and reduce residual stress in the cavity. The stent has a number of uniformly-distributed injecting gates, which are contributive to the transfer of pressure, increasing the material supplement, reducing the shrinkage of the flat plate, and decreasing the warpage. Therefore, the effect of melt temperature on residual stress and warpage is the comprehensive result of these above-mentioned factors.

**Figure 12:**
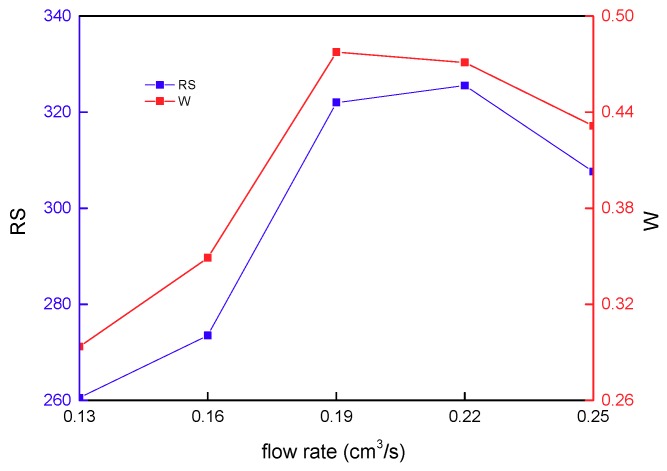
The effect of packing pressure on the residual stress and warpage.

The influence of flow rate on the residual stress and warpage of the PLA stent is presented in Figure 12, showing a non-linear relationship obviously. The flow rate of the melt in the cavity is basically controlled by the flow rate of the injection. If the cavity structure is the same as other conditions, a relatively small flow rate would cause the melt to flow slowly in the cavity, and the melt compressed at the injecting gate can be fully loosened during the slow flow process. Furthermore, the melt has a relatively small uniformly-distributed residual stress in the follow-up packing and cooling process, which makes the warpage very small. With the increase in flow rate, the quicker the melt flows in the cavity, the bigger the flow and shear rate of the melt is, and the higher its shear stress becomes. Typically, the above-mentioned facts result in the higher residual stress of a stent, and even serious warpage. With the further increase in flow rate, the flow and shear rate of the melt increases. At the same time, melt viscosity also decreases to make the melt easily flow, and allow the cavity to be filled well, which can reduce residual stress and warpage. Apparently, the flow rate can affect the looseness and viscosity of the melt; weighing the proportion of these two by adjusting the flow rate can effectively reduce residual stress and warpage. Indeed, a relatively small flow rate correlates to longer injection molding time, while a relatively large flow rate correlates to greater injection pressure. In the actual molding process, equipment and production efficiency should be considered. Furthermore, as for various types of materials, the effect of the flow rate on residual stress and warpage is relatively complex, because the mold-filling rate of the melt depends on the flow rate, while the effect of the mold-filling rate on plastic product properties is also relatively complex, which is correlated to the type of material.

**Figure 13:**
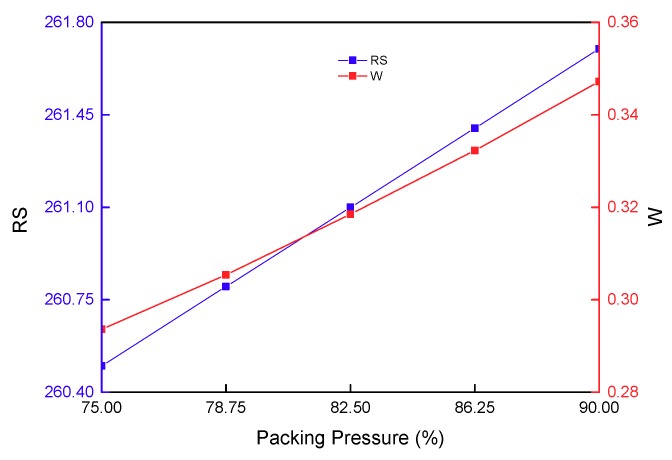
The effect of packing pressure on the residual stress and warpage.

The effect of packing pressure on the residual stress and warpage of a stent is presented in Figure 13. As for the thin-wall stent structure of the PLA material, merely relatively low packing pressure can ensure the relatively small warpage and residual stress. As for thin-walled parts, a relatively high packing pressure increases residual stress, causing non-uniform shrinkage, thereby resulting in a relatively larger warpage. Indeed, plastics and products with a relatively large viscosity, and have relatively high requirements in small wall thickness, a long process and high accuracy, needs a relatively high packing pressure. The present study considers these from the perspective of the optimal design.

## 4. Discussion

As for the biodegradable PLA polymeric stent, the shortage of an accurate and effective manufacturing method is a potential limit to the development of polymeric stents. A PLA stent, which has low mechanical strength and an extremely tiny volume, is a typical precision thin-walled product. Due to its complex stent structure and tiny local size, the microinjection molding stent is greatly different from products manufactured by conventional microinjection molds. The stent product has a large length-to-diameter ratio, and a relatively low surface continuity, which influences the flowability of the polymeric melt in the cavity. Therefore, it would be difficult to completely fill the cavity during the experiment process. This is one of main difficulties of microinjection molding. In addition, the stent can easily form weld marks on the surface, and the number of the weld marks is expected to be more than that of conventional injection molding products.

In the design process, injection molding process parameters should be repeatedly adjusted to meet the quality requirements of plastic parts. The unreasonable parameters in the injection molding process, such as mold temperature, melt temperature, flow rate, and packing pressure, can result in serious residual stress and warpage of the stent. A relatively large residual stress in the cavity can result in difficulties during the demolding process, and even damage the stent during the demolding process. Furthermore, a relatively large warpage can greatly influence the product’s use properties, and even its normal use.

In the present study, in order to reduce residual stress and warpage in the polymeric stent injection molding process, the study on the effect of process parameters, such as mold temperature, melt temperature, flow rate and packing pressure, can provide guidance in selecting the process parameter combination during the injection molding process. The scope of the studied process parameters are as follows: mold temperature (80, 90), melt temperature (190, 205), flow rate (0.13, 0.25), and packing pressure (75%, 90%). Therefore, the purpose of the optimal design in the stent injection molding process was to maximize the residual stress and warpage of the stent, thereby reducing the difficulties of demolding and the use properties of the product. Compared to the results of the comparable design, residual stress decreased by 20.3%, while warpage decreased by 44.6%. These apparently contributed to enhancing the molding quality of the stent. It should be acknowledged that the present study came to this conclusion based on the PLC material, without a setting of a cooling waterway, and without considering the effect of the actual cooling process. When the stent materials were different, or the mold structure was different from that in the present study, this would result in the optimal results of the stent’s different process parameters. Of course, when investigating polymer stent of different materials or different structures, the optimization results of the injection molding process will be different. This research highlights how the surrogate model can be established and used to optimize the stent injection molding process in order to demonstrate that the process can be designed and improved through this important approach.

## 5. Conclusions

The present study presents a multi-objective optimization method combined with the Kriging surrogate model to improve PLA stent injection molding process parameters, with the aim of enhancing the injection molding quality of micro absorbable polymeric stents. The Kriging surrogate model coupled with DOE methods was adopted to establish the approximate relationship between objective function and design variables. These results show that the proposed optimization method could be effectively used for the microinjection molding process. These results indicate that it is very useful based on the proposed optimization method to improve the molding quality during the microinjection molding process. This exhibits a novel methodology for the injection molding process design of polymer stents, representing a new research direction. The combination of this optimization method and experimental verification can play an important role in the process design of injection molding stents.

## Figures and Tables

**Table 1:** The range of the process parameter.

Stent	$T_{mold}$ (°C)	$T_{melt}$ (°C)	$v_{flow}$ (cm^3^/s)	$P_{pack}$ (%)
Lower limit	80	190	0.13	75
Upper limit	90	205	0.25	90

**Table 2:** Comparison of the two design results.

Stent	$T_{mold}$ (°C)	$T_{melt}$ (°C)	$v_{flow}$ (cm^3^/s)	$P_{pack}$ (%)	RS	W
Comparable design	85	197.5	0.19	82.5	326.9	0.5304
Optimal design	87.5	205	0.13	75	260.5	0.2936

## References

[1] Onuma Y., Serruys P.W. (2011). Bioresorbable scaffold: the advent of a new era in percutaneous coronary and peripheral revascularization?. Circulation.

[2] Clarke G., Mulvihill H., Duffy A. (2008). Bioresorbable Stent and Method of Making. U.S. Patent.

[3] Liu D.L., Shen C.Y., Liu C.T., Xin Y., Sun L. (2011). Investigation of mold temperature affecting on shrinkage of rapid heat cycle molding plastic part. Adv. Mater. Res..

[4] Duo Y. (2014). Effect of micro-injection molding process parameters for warpage in Micro Plate Devices. Adv. Mater. Res..

[5] Jiang J., Wang S., Sun B., Ma S.J., Zhang J.M., Li Q., Hu G.H. (2015). Effect of mold temperature on the structures and mechanical properties of micro-injection molded polypropylene. Mater. Des..

[6] Singh G., Verma A. (2017). A Brief Review on injection moulding manufacturing process. Mater. Today.

[7] Annicchiarico D., Alcock J.R. (2014). Review of factors that affect shrinkage of molded part in injection molding. Mater. Manuf. Process..

[8] Mohan M., Ansari M.N.M., Shanks R.A. (2017). Review on the effects of process parameters on strength, shrinkage, and warpage of injection molding plastic component. Polym. Plast. Technol. Eng..

[9] Shen C., Wang L., Li Q. (2007). Optimization of injection molding process parameters using combination of artificial neural network and genetic algorithm method. J. Mater. Process. Technol..

[10] Gheorghe O.C., Florin T.D., Vlad G.T., Gabriel D.T. (2014). Optimization of micro injection molding of polymeric medical devices using software tools. Procedia Eng..

[11] Kitayama S., Natsume S. (2014). Multi-objective optimization of volume shrinkage and clamping force for plastic injection molding via sequential approximate optimization. Simul. Model. Pract. Theory.

[12] Oliaei E., Heidari B.S., Davachi S.M., Bahrami M., Davoodi S., Hejazi I., Seyfi J. (2016). Warpage and shrinkage optimization of injection-molded plastic spoon parts for biodegradable polymers using taguchi, ANOVA and artificial neural network methods. J. Mater. Sci. Technol..

[13] Dang X.P. (2014). General frameworks for optimization of plastic injection molding process parameters. Simul. Model. Pract. Theory.

[14] Kashyap S., Datta D. (2015). Process parameter optimization of plastic injection molding: A review. Int. J. Plast. Technol..

[15] Kitayama S., Yamazaki Y., Takano M., Aiba S. (2018). Numerical and experimental investigation of process parameters optimization in plastic injection molding using multi-criteria decision making. Simul. Model. Pract. Theory.

[16] Krige D.G. (1951). A statistical approach to some basic mine valuation problems on the Witwatersrand. J. South. Afr. Inst. Min. Metall..

[17] Spina R., Spekowius M., Hopmann C. (2016). Multiphysics simulation of thermoplastic polymer crystallization. Mater. Des..

[18] Gao Y.H., Wang X.C. (2009). Surrogate-based process optimization for reducing warpage in injection molding. J. Mater. Process. Technol..

[19] Li H.X., Wang X.Y., Wei Y.B., Liu T., Gu J.F., Li Z., Wang M.J., Zhao D.Y., Qiao A., Liu Y.H. (2017). Multi-Objective Optimizations of biodegradable polymer stent structure and stent microinjection molding process. Polymers.

[20] Joseph V.R., Hung Y. (2008). Orthogonal-maximin Latin hypercube designs. Stat. Sin..

